# Does the Stent Retriever Placement in the Division of Middle Cerebral Artery Affect the Recanalization Success in M1 Occlusions?

**DOI:** 10.5152/eurasianjmed.2022.20281

**Published:** 2022-02-01

**Authors:** Nihat Sengeze, Semih Gıray

**Affiliations:** 1 Department of Neurology, Süleyman Demirel University Hospital, Isparta, Turkey; 2 Departmant of Neurology, Gaziantep University Hospital, Gaziantep, Turkey

**Keywords:** Mechanical thrombectomy, middle cerebral artery occlusion, stent retriever placement, recanalization success

## Abstract

**Objective:** The location of arterial occlusions can be predictive in the prognosis and endovascular treatment of acute stroke patients. We aimed to determine if the location of the stent retriever being on the superior or inferior division of the middle cerebral artery has an effect on the success and clinical outcomes of recanalization in middle cerebral artery M1 occlusion.

**Materials and Methods:** Data were generated for the period from May 2015 to January 2019. Divisions of middle cerebral artery were assigned to the 2 groups as superior and inferior divisions according to the anatomical classification. The dominant trunk of the artery was assessed on the last angiogram image.

**Results:** We eventually included 81/90 patients (mean age: 62 ± 13.5; 63% [51/81] female; mean National Institutes of Health Stroke Scale rating: 16.3 ± 3.6) treated with thrombectomy. The branches of the middle cerebral artery were as follows: 40 (49.4%) co-dominant, 22 (27.2%) inferior, and 19 (23.5%) superior division dominant. The stent retriever was placed in the dominant trunk in 22/41(53.7%) cases at first pass. When stent retriever was placed in the dominant middle cerebral artery trunk, the rate of successful recanalization was very high with the first pass of thrombectomy (*P* < .001).

**Conclusion:** Stent retriever placement within the superior or inferior middle cerebral artery trunk does not have an effect on the success rate of recanalization; however, its placement in the dominant trunk can increase the chance of complete recanalization to be early.

**Main Points**

- In recent years, endovascular treatment has been the most attractive and life-saving treatment in stroke treatment all over the world.
- The most important step for mechanical thrombectomy in stroke treatment is to provide successful recanalization with the first pass. This is called the “first-pass effect.’’
- Finding a simple and reliable parameter that will affect the “first-pass effect’’ in mechanical thrombectomy makes an important contribution to stroke treatment.
- There are often 2 branches of middle cerebral artery to place the stent retriever to recanalize middle cerebral artery occlusion. The question is what is the significance of this choice for recanalization success?
- This article shows that what matters is not the inferior or superior division but the dominant one.

## Introduction

The middle cerebral artery (MCA) is the most developed terminal branch of the carotid artery. Middle cerebral artery has 2 main segments: the proximal and the distal. The proximal segment (M1) extends from the internal carotid artery to the limen of the insula, between the temporal and the frontal lobes. The distal segment (M2) extends from the limen of the insula to the terminal point of the MCA. From the main trunk of M2, leptomeningeal branches arise, singular or together with the common trunk, and diverge throughout the insula.^1^ When the MCA arises cortically, it can originate from the superior, middle, or inferior trunk. The inferior trunk usually gives rise to the temporal arteries in both bifurcation and trifurcation. If bifurcation occurs, the superior trunk typically gives rise to the orbitofrontal, prefrontal, precentral, and central ­arteries.^2^ Fisher in 1938 described M1 as the sphenoidal segment that could include or not include the main trunk bifurcation and the M2 as the insular segment. However, with the initiation of mechanical thrombectomy operators, in an arbitrary manner, they decided to name M1 as the main trunk and M2 the divisions.

Since MCA occlusions are relatively more frequent, the knowledge of anatomical variations of the MCA provides ease in both surgical and endovascular treatments.^3^

The success of recanalization at first pass with mechanical thrombectomy (MT) is still 50%. We wanted to determine whether the stent retriever (SR) placement in the superior or inferior branch of MCA could affect the recanalization success or not. For this reason, we examined the anatomical structures of MCA and the effect of SR placement on the recanalization success.

The purpose of this study was also to provide information regarding the best place of SR in the MCA to potentially improve the recanalization result.

## Materials and Methods

### Patients

An analysis was performed to determine patients who underwent MT retrospectively. Digital subtraction angiography (DSA) and baseline parameters were prospectively analyzed from the database. Patients (18-80 years) with MCA M1 occlusions and proper DSA documentation were included in the study. In this procedure, superior and inferior divisions of M1 were detected after the recanalization view in post-thrombectomy of DSA.

Patients were excluded from the study if they had an occlusion of artery other than MCA M1, were treated with only 1 aspiration technique (a direct aspiration first pass technique technique (ADAPT)), and had no decided superior and inferior branches anatomically. The successful recanalization was defined with a modified thrombolysis in cerebral infarction (mTICI) score (mTICI ≥ 2b). According to the Declaration of Helsinki guidelines, ethics approval was received for the acquisition of patient data from the local ethics committees with the date April 3, 2019 and number 2019/160. Informed consent was obtained from all patients in writing before the procedure and this information was stated in the permission of the ethics committee.

**Figure 1.**
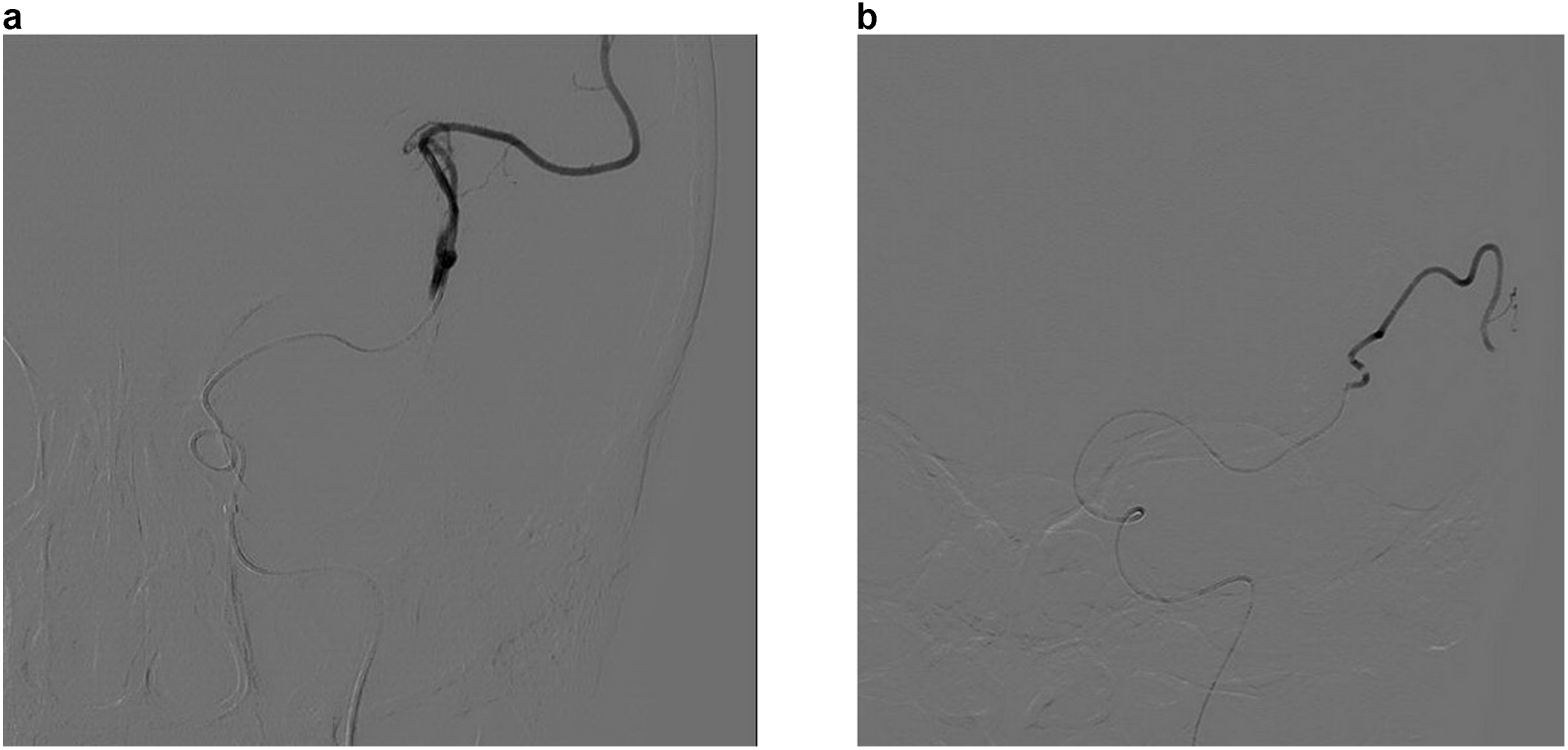
(a) Determination of superior branch by microcatheter injection. (b) Determination of inferior branch by microcatheter injection.

Evaluation of MCA anatomy was performed using the following steps:

- Middle cerebral artery M1 division point was determined based on the place where the main insular trunks converge on anterior–posterior plane. Then the division point of the M1 artery was verified on lateral planes on the DSA image.
- Domination of MCA divisions (superior or inferior trunk) was evaluated on anterior–posterior plane on the final DSA image. No domination was determined when there was no significant difference in diameter of MCA trunks (less than difference of 20% in diameter) after M1 division. In evaluation of trunk domination, the area of vascularization was also factored in (superior MCA trunk; orbitofrontal and posterior parietal areas—inferior MCA trunk; temporal, temporo-occipital, and angular areas) vascular supply that tested with microcatheter contrast injection. Digital subtraction angiography images of MCA anatomy evaluation with microcatheter injection are presented on Figure 1.

Divisions of MCA were assigned to the 2 groups as superior and inferior divisions according to the anatomical classification.

### Angiographic Analysis

**Figure 2.**
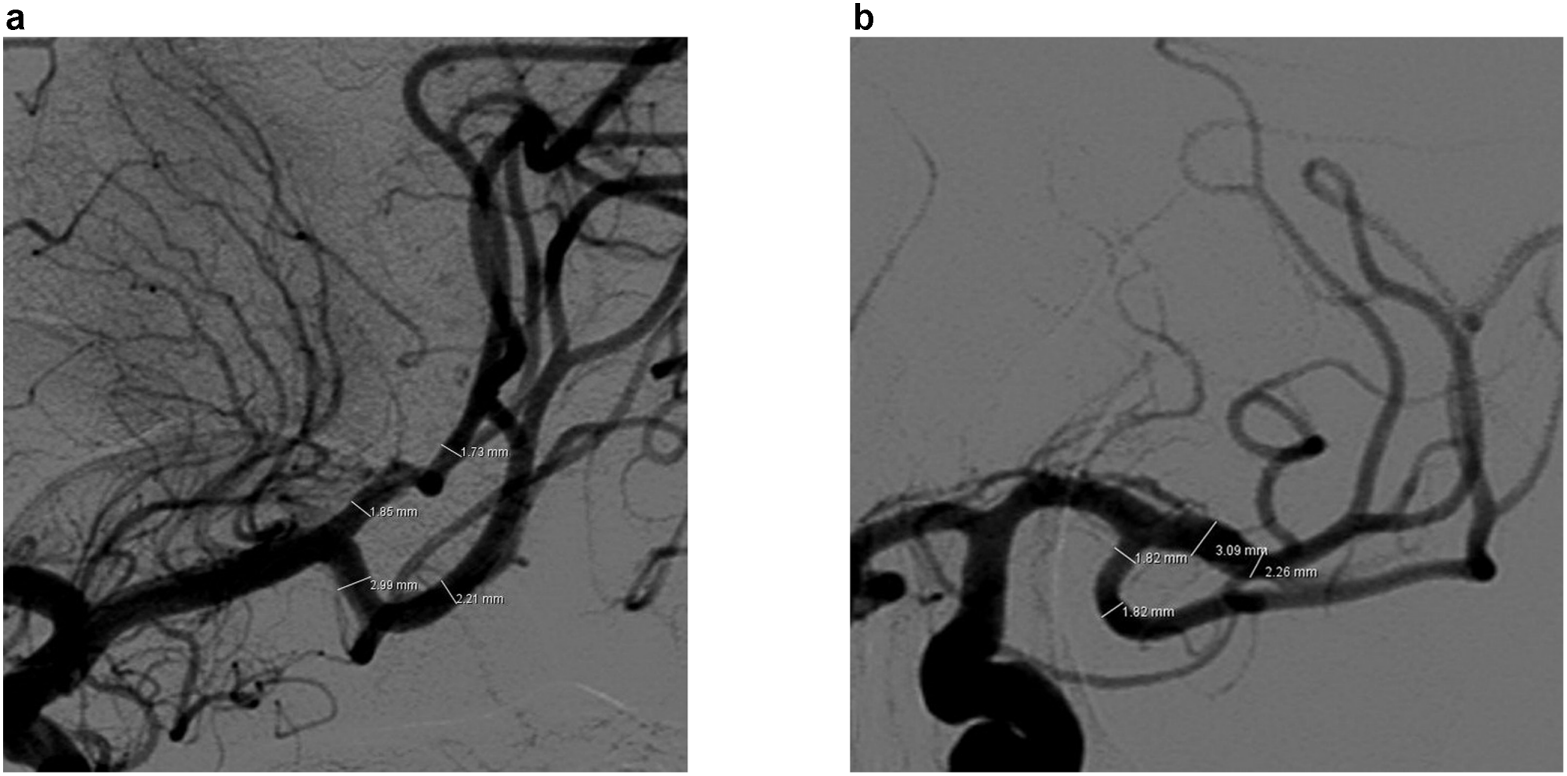
(a) Determination of the dominance of superior branch by measurements. (b) Determination of the dominance of inferior branch by measurements.

**Figure 3.**
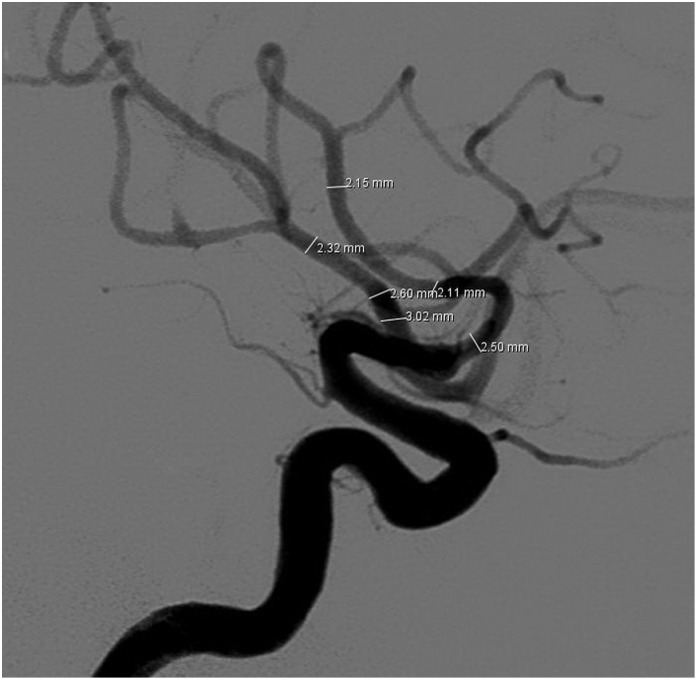
Lateral view measurements in DSA to determine the dominant branch.

The DSA series after the MT were re-evaluated with 2 other interventional neurology experts who were blinded to the clinical results and the demographic data. The MCA trunk of each patient (superior or inferior) was evaluated by microcatheter injection prior to the SR attempt. The allocation of the navigated MCA division (superior or inferior trunk) was decided on the last DSA image in cases of successful recanalization. If there was a difference of 20% between the arterial diameter measurements of superior and inferior divisions, the highest one was considered as dominant. The dominant trunk (DT) of artery was assessed on the last angiogram image (Figure 2, 3).

### Statistical Analysis

Frequency, percentage (%), and mean ± standard deviations (mean ± SD) were given as descriptive statistics. Descriptive statistics are shown as mean and SD if normally distributed and as median and Interquartile range (IQR) if not. The Mann–Whitney *U* test and the Fisher test were performed for assessing statistical differences between groups. Statistical significance was defined as *P* ≤ .05. Chi-square test was used to assess relation between categorical variables. Statistical analysis was performed with Statistical Package for the Social Sciences software for Windows version 24.0 (IBM SPSS Corp.; Armonk, NY, USA) and a *P*-value < .05 was accepted as statistically significant.

Groups were formed according to the width of the artery measured to determine the dominance. The success of recanalization according to the dominant artery selection in SR location was compared with the chi-square method.

## Results

From a total of 90 patients, we eventually included 81 patients (mean age 62 ± 13.5 years; 63% [51/81] female; mean National Institutes of Health Stroke Scale rating 16.3 ± 3.6, mean Alberta stroke programme early CT score (ASPECT) 9 ± 1.2) treated with SR-based thrombectomy. We excluded 5 patients as we could not decide on the placement of the SR anatomically and not reach the SR in the superior or inferior division at the end of the endovascular therapy. Also, 4 other patients were excluded because of successful recanalization without the use of the SR (aspiration and intra-arterial thrombolysis). Intravenous tissue plasminogen activator (TPA) was used in 17 (21%) of the patients before the endovascular procedure and intra-arterial TPA was used in 58 (71.6%) of them during the endovascular procedure. Of the 81 patients, there were 41 (50.6%) with right-sided hemiparesis of stroke and 59 (72.8%) were treated with successful recanalization (mTICI 2c-3) at the end of the endovascular therapy. After 3 months, 24 (19.6%) patients achieved mRS of 0-2, 40 (49.4%) achieved mRS of 0-3, and 31 (38.3%) patients died (Table 1). The causes of patient mortality were as follows: 21 (68%) due to cerebral edema as a result of cerebral infarction or cerebral hemorrhage, 5 (16%) due to sudden cardiac arrest, and 5 (16%) due to aspiration pneumonia.

After the endovascular procedure, 7 (8.6%) patients had symptomatic intracranial hemorrhage as subarachnoid hemorrhage, 5 (6.1%) had superficial hematoma in the femoral artery region, and 1 (1.2%) had a pseudoaneurysm in the femoral artery.

When the MCA superior and inferior divisions were examined in terms of arterial dominance, 40 (49.4%) patients were co-dominant, 22 (27.2%) were inferior division dominant, and 19 (23.5%) were superior division dominant. Only 3 of 81 (3.7%) patients had trifurcation of MCA division, and if the SR placement was in the superior and middle trunk, they were accepted as superior division placement.

Out of the 81 patients who had a first-pass thrombectomy, 54 (66.7%) had the microcatheter placed in the superior trunk and 27 (33.3%) had it placed in the inferior trunk. Of the 37 patients who went through a second pass of thrombectomy, 16 (43%) had the microcatheter placed in the superior and 21 (57%) had it placed in the inferior trunk. Finally, of the 14 patients who had third-pass thrombectomy, 8 (57%) had the microcatheter placed in the superior and 6 (43%) had it placed in the inferior trunk (Table 2).

The correlation with SR placement in divisions of MCA and recanalization of thrombectomy were also studied. The results are shown in Table 3 and 4.

Stent retriever was placed within the DT in 22 (53.7%) cases at the first pass of thrombectomy. The rate of successful recanalization in the first-pass thrombectomy was higher when the SR was placed in the DT versus the non-dominant trunk [mTICI 2b-3: 19/22 (87%) vs 3/19 (16 %); *P* < .001, mTICI 2c-3: 14/22 (64%) vs 1/19 (%5); *P* < .001, and mTICI 3: 9/22 (41%) vs 1/19 (%5); *P* = .008].

Stent retriever was placed within the DT in 13 (72.2%) cases at the second pass of thrombectomy. The rate of successful recanalization in second-pass thrombectomy was higher when the SR was placed in the DT versus the non-dominant trunk [mTICI 2b-3: 10/13 (77%) vs 1/5 (20%); *P* = .026, mTICI 2c-3: 7/13 (54%) vs 1/5 (20%); *P* = .196, mTICI 3: 2/13 (15%) vs 1/5 (20%); *P* = .814].

The number of patients for the third pass was significantly reduced. Therefore, statistical analysis was not made.

## Discussion

In our study, when the MCA superior and inferior divisions were examined in terms of arterial dominance, 40 (49.4%) patients were co-dominant, 22 (27.2%) were inferior division dominant, and 19 (23.5%) were superior division dominant. The trunk co-dominance of MCA in our study was also similar to the literature in anatomical studies.^1-4^ There were 41 patients within the 2 groups for the dominant branch. This was a very small study population to achieve a strong conclusion but it can still give us an idea.

There have been some studies on MCA branching. Jeyakumar and Veerapandia^4^ noted that MCA bifurcated into superior and inferior trunk in 22 cases. Middle cerebral artery trifurcated into the superior, middle, and inferior trunks in 8 (9.8%) cases. Similarly in another study, Maslehaty et al^5^ analyzed data of 300 patients and bifurcation was observed in 72% of them while trifurcation was observed in 12% and false bifurcation in 16% of patients. Therefore, we often see the divisions of MCA as superior and inferior branches. There is also anatomically superior and inferior branch dominance or co-dominance can be observed. Umansky et al^6^ investigated that the inferior trunk was dominant in 32%, the superior in 28% of patients, whereas they were equal in 18% (multiple trunks of various diameters in 22%) of the patients.

Stent retriever-based MT is the most current approach in the treatment of large vessel occlusions seen in the anterior cerebral circulation due to the stroke.^7^ Defining the segments of MCA can be useful in deciding the eligibility of the patient for interventional treatment. It is still debated what should be considered as normal MCA anatomy. However, most of the authors consider both bifurcation and trifurcation as normal anatomy.^3^ Mechanical thrombectomy is of quite effective clinical value in selected patients with acute stroke caused by large vessel occlusion based on recent endovascular therapy trials. Despite this treatment, almost half of the patients did not have acceptable clinical outcomes.^8^

As we stated in our hypothesis, in the endovascular treatment of acute MCA M1 obstructions, the MCA branch in which the SR will be placed may affect the success and duration of recanalization. In our study, we can state that the SR placement in the inferior or superior trunk may not influence the rate of recanalization success. Its placement in the DT can increase the rate of complete (mTICI 3) recanalization. Maus et al.^9^ also investigated this issue in MCA M1 (89 patients) occlusions with a study. Stent retriever placement in the DT occurred in 40 (53%) of 76 patients in their study. The rate of complete recanalization in first-pass thrombectomy was higher when the SR was located within the DT versus the non-dominant trunk. Maus et al^9^ stated in their research that the first-pass complete reperfusion (mTICI 3) was associated with a larger internal diameter of the artery (superior or inferior trunk) used for SR location on angiograms. This was different from our study regarding the outcome. Another noteworthy concept here is that not every recanalization will mean reperfusion. In the study conducted by Maus et al^9^, arterial diameter differences were examined numerically. In this study, the mean MCA superior and inferior division widths were given as 1.4 mm and 1.18 mm, respectively. In fact, we identified the co-dominant artery if the diameter difference of the branches was lower than 20%. However, in our study, we chose to determine the diameter difference of 20% and above, which may be clinically significant, as the dominant artery. Thus, in our study, we showed that the SR location being in the dominant division rather than in the superior or inferior branch increases the success of recanalization.

Quereshi et al^10^ hypothesized that if the recanalization was successful with fewer SR passes, there were higher rates of recanalization and good clinical results in patients with MCA M1 occlusions than the divisions of MCA (superior or inferior trunk) occlusions. In the final analysis of the 32 patients in their study, 11 had main trunk MCA occlusions and 21 had division of MCA occlusions. When the placement of the SR was analyzed with comparison of the recanalization in the dominant versus non-dominant division, 3 passes of the main M1 trunk MCA occlusions per patient were required. Recanalization rates in the dominant division were statistically higher (*P* = .02). In that study, there were only 21 passes in 11 patients with main trunk MCA occlusion. Statistically, we also found high recanalization rates in favor of the dominant division as we conducted this study in a larger (n = 81) group of patients with main trunk MCA occlusions.

Although the efficacy of SR MT for acute ischemic stroke with large-vessel occlusion was proven, it had a potential risk of vessel wall injury because of continuous radial force against the vessel wall.^11^ In this respect, the location of the stent for the first-pass recanalization success is quite important to avoid similar complications. Injection of contrast material through a microcatheter before the stent opening should be considered to see which artery we are in and to determine the size of a suitable SR.

Finally, location of the SR is very important for the successful recanalization for the first pass. The less endothelial vascular trauma is more likely to occur with a single-device pass versus multiple passes.^12^ In a recently published article that draws attention to the importance of vessel diameter for the first-pass effect, Srivatsa et al^13^ showed that a larger M1 diameter increased recanalization success with the first pass. Another method used to obtain a first-pass effect may be the dual SR technique. Asadi et al^14^ and Patro et al^15^ claimed that the dual SR technique might be particularly helpful for refractory clots involving arterial bifurcation, which are resistant to multiple passes of a single SR during MT. Hence, achieving complete revascularization with a single pass should be the primary angiographic goal as thrombectomy device design continues to improve. As they have stated, the application of dual SR may further increase the risk of vessel injury by increasing exposure of more metals to the vessel endothelium during retrieval maneuvers. In this respect, SR location becomes more important when a single SR is used. Based on our endovascular treatment experience, advancement of microwire to MCA by J-shape technique and contrast injection after microcatheter placement may lead us in the selection of dominant trunk. On the other hand, placing the wire toward the parietal lobe (intermediate trunk) in that way will most likely cause the SR to fall into the larger division which is most likely to be the dominant division.

There are some strengths as well as limitations of our study. The study had a small number of patients who were excluded due to use of the aspiration technique for recanalization and not being able to determine the superior and inferior branches anatomically. On the other hand, recanalization, such as using the different types of SR devices which were not grouped, were included in the study. This made it more difficult to determine the precise effect of this approach. Furthermore, other factors might have influenced the success rates of thrombectomy. For example, thrombus structure and etiology of thrombus were the other factors to be investigated.

Our findings also suggest that the position of the SR in patients treated with MT due to a MCA M1 occlusion affects the recanalization results. Our data indicates that SR placement within the MCA division (superior or inferior trunk) may not affect the rate of recanalization success. Its placement in the DT can increase the rate of complete recanalization (mTICI 3) with fewer SR passes. Nevertheless, larger prospective studies are required to validate the usefulness and safety of this strategy and its ability to improve clinical outcomes. Until then, this technique may be used with checking the artery after microcatheter contrast injection to protect against potential risks of dual stent thrombectomy and multiple passes of thrombectomy.

In conclusion, stent retriever placement within the superior or inferior MCA trunk does not have an effect on the success rate of recanalization; however, its placement in the dominant trunk can increase the chance of complete recanalization early.

## Figures and Tables

**Table 1.** Baseline and Clinical Characteristics of All Patients

	Baseline and Clinical Characteristics (n = 81)
N (%)
Sex, female	51 (63)
Stroke side, right	41 (50.6)
IV-TPA	17 (21)
IA-TPA	58 (71.6)
Antiplatelet drug history	15 (18.5)
Atrial fibrillation	31 (38.3)
Hyperlipidemia	31 (38.3)
Hypertension	59 (72.8)
Smoking	18 (22.2)
Diabetes mellitus	21 (25.9)
Coroner artery disease	19 (23.5)
Stroke at history	4 (4.9)
mRS (0-3)	40 (49.4)
Mortality in 3 months	31 (38.3)

mRS: modified Rankin Scale, IV-TPA, intravenous tissue plasminogen activator; IA-TPA, intraarterial tissue plasminogen activator.

**Table 2.** Stent Retriever Placement for the Division of Middle Cerebral Artery

Number of Pass	Inferior Trunk (n %)	Superior Trunk (n %)
First pass	27/81 (33)	54/81 (67)
Second pass	21/37 (57)	16/37 (43)
Third pass	6/14 (43)	8/14 (57)

**Table 3.** Recanalization Results Dependent on Stent Retriever Placement in M1 Occlusion at First Pass of Thrombectomy

Recanalization Success	Inferior Trunk	Superior Trunk	P*	Dominant Trunk	Non-Dominant Trunk	P*
mTICI 2b-3	17/27 (63)	25/54 (60)	0.157	19/22 (87)	3/19 (16)	<.001
mTICI 2c-3	11/27 (41)	19/54 (35)	0.625	14/22 (64)	1/19 (5)	<.001
mTICI 3	9/27 (33)	13/54 (24)	0.377	9/22 (41)	1/19 (5)	.008

^*^Chi-square test, mTICI: modified thrombolysis in cerebral infarction.

**Table 4.** Recanalization Results Dependent on Stent Retriever Placement in M1 Occlusion at Second Pass of Thrombectomy

Recanalization Success	Inferior Trunk	Superior Trunk	*P* ^*^	Dominant Trunk	Non-Dominant Trunk	*P* ^*^
mTICI 2b-3	13/21 (62)	9/16 (56)	0.729	10/13 (77)	1/5 (20)	.026
mTICI 2c-3	4/21 (19)	7/16 (44)	0.103	7/13 (54)	1/5 (20)	.196
mTICI 3	2/21 (9.5)	3/16 (19)	0.416	2/13 (15)	1/5 (20)	.814

^*^Chi-square test, mTICI: modified thrombolysis in cerebral infarction.
